# Taxon-specific contributions of microeukaryotes to biological carbon pump in the Oyashio region

**DOI:** 10.1093/ismeco/ycae136

**Published:** 2024-11-04

**Authors:** Qingwei Yang, Yanhui Yang, Jun Xia, Hideki Fukuda, Yusuke Okazaki, Toshi Nagata, Hiroyuki Ogata, Hisashi Endo

**Affiliations:** Bioinformatics Center, Institute for Chemical Research, Kyoto University, Gokasho, Uji, Kyoto, 611-0011, Japan; Atmosphere and Ocean Research Institute, The University of Tokyo, 5-1-5, Kashiwanoha, Kashiwa, Chiba 277-8561, Japan; Bioinformatics Center, Institute for Chemical Research, Kyoto University, Gokasho, Uji, Kyoto, 611-0011, Japan; Atmosphere and Ocean Research Institute, The University of Tokyo, 5-1-5, Kashiwanoha, Kashiwa, Chiba 277-8561, Japan; Bioinformatics Center, Institute for Chemical Research, Kyoto University, Gokasho, Uji, Kyoto, 611-0011, Japan; Atmosphere and Ocean Research Institute, The University of Tokyo, 5-1-5, Kashiwanoha, Kashiwa, Chiba 277-8561, Japan; Bioinformatics Center, Institute for Chemical Research, Kyoto University, Gokasho, Uji, Kyoto, 611-0011, Japan; Bioinformatics Center, Institute for Chemical Research, Kyoto University, Gokasho, Uji, Kyoto, 611-0011, Japan

**Keywords:** microeukaryotes, biological carbon pump, sinking particles, suspended particles, marine snow catcher, DNA metabarcoding

## Abstract

Microeukaryotes are critical components of sinking particles contributing to carbon export from the surface to deep oceans. However, the knowledge of the sinking microeukaryotic communities and their dynamics is currently limited. In this study, we applied 18S rRNA gene metabarcoding to investigate the microeukaryotic communities in sinking and suspended particles distinguished by marine snow catchers during spring in the Oyashio region. Sinking particles displayed distinct communities and lower diversity than suspended particles. The community compositions of the sinking particles varied with depth, suggesting that microeukaryotes were selectively disaggregated or decomposed during settling. Prymnesiophyceae and diatoms were effectively removed, as indicated by their decreased abundance in sinking particles at increasing depths. Conversely, Dinophyceae maintained a higher abundance in sinking particles across depths, indicating resistance to disaggregation and decomposition. Spirotrichea and heterotrophic Dinophyceae were enriched in sinking particles, while marine stramenopiles groups were enriched in suspended particles. The heterotrophs in the deeper layers were mainly transported from the surface layers by increasing their relative abundance towards deep layers, indicating that they contributed to the transformation processes of sinking particles. Overall, our results demonstrate the functional differences among microeukaryotes in the biological carbon pump.

## Introduction

The sinking of particulate organic carbon (POC) is a major driver of the oceanic biological carbon pump (BCP), a process through which biologically fixed carbon is transferred from the euphotic layer to the deep ocean [1]. Global estimates of the sinking POC flux out of the surface mixed layer range from 5 to 20 Gt C yr^−1^ [2]; this ultimately influences the air–sea carbon dioxide exchange and Earth’s climate [3]. Among the numerous types of particulate constituents of sinking POC, microeukaryotes, including photosynthetic and heterotrophic protists, determine the structure, sinking velocity, and chemical composition of sinking POC, thereby influencing the magnitude and efficiency of the BCP [4–6]. Therefore, examining the taxonomic composition and variability of the microeukaryotic assemblages in sinking particles is essential.

Microeukaryotes in sinking particles have been conventionally collected using sediment traps and examined using light microscopy and biomarker pigment analysis [7, 8]. Silicified or calcified phytoplankton, such as diatoms and coccolithophorids, are often abundant in sinking particles, suggesting that their mineral cell walls (frustules or plates) function as ballasts to enhance the settlement of aggregates containing these cells or their remnants [9]. Nonetheless, these techniques have limited taxonomic resolution and fail to identify protists lacking mineral cell walls, especially when small and non-pigmented (i.e. heterotrophs). Recent advances in environmental DNA-based approaches, such as the 18S rRNA gene metabarcoding method [10–17], offered comprehensive information on community composition, improving our understanding of the role of microeukaryotes in the export of carbon to the deep ocean. For instance, Amacher et al. (2009) revealed that small phytoplankton, such as prasinophytes and uncalcified prymnesiophytes, dominated sediment trap particles despite the high abundance of diatoms in the water column [10]. Gutierrez-Rodriguez et al. (2019) showed active consumption of sinking aggregates, which are dominated by radiolarians, by heterotrophs [12]. Durkin et al. (2022) demonstrated that the varying relative contribution of phytoplankton in sinking particles across taxa, and the degradation states also varied across oceanic regions [17]. Time-series sediment trap studies at abyssal depths documented the occurrence of various eukaryotic lineages of protists originating from surface layers [13, 15].

Despite the growing knowledge of microeukaryotic assemblages associated with sinking particles, some critical questions remain unresolved. One such question is whether certain microeukaryotes are preferentially associated with sinking rather than suspended particles and are more efficiently transferred from the sunlit layer to abyssal depths. The sinking POC flux is strongly attenuated through the mesopelagic layer (200–1000 m), leaving less than 10% of the exported POC available for consumption in the bathypelagic layer and abyssal sediments [18, 19]. Several biological and physical processes have been proposed to explain this depth-dependent POC flux attenuation, including microbial degradation and solubilization [20], zooplankton consumption, and fragmentation due to the turbulence created by zooplankton swimming and feeding activities [21]. Although the relative importance of different mechanisms remains elusive, microeukaryotic cells that are more resistant to POC flux attenuation processes (disaggregation or decomposition) could contribute more substantially to carbon transfer to deeper layers, thereby enhancing the efficiency of the BCP. Conversely, easily disaggregated, or degraded microeukaryotes could contribute to carbon remineralization in the mesopelagic layer.

Recently, a large sedimentation chamber [namely, the Marine Snow Catcher (MSC)] was used to differentiate microbial assemblages in sinking and suspended particles [14, 22–25]. Of note, the composition of microeukaryotic assemblages differed between sinking and suspended particles in the Scotia Sea [14]. In the upper mesopelagic layer, chain-forming diatoms dominated the sinking particle assemblages, whereas prymnesiophytes were abundant in suspended particles, implying that diatom-enriched particles are more efficiently transferred to the mesopelagic layer than prymnesiophytes-enriched ones. Although this previous study provided valuable insights into the functional differences among microeukaryotic taxa in the regulation of the BCP, the data were limited to the upper mesopelagic layer, inevitably hampering the assessment of microeukaryotic dynamics throughout the mesopelagic water column.

In the current study, we employed the MSC to characterize the microeukaryotic community associated with sinking and suspended particles and their changes during sedimentation through the mesopelagic water column. Sampling was conducted during spring blooms in the Oyashio waters off Hokkaido in the western North Pacific Ocean [26]. This oceanic region is known for its strong POC flux [27] and highly efficient BCP [28] during blooms. Earlier sediment trap observations suggested that diatoms in this area play a key role in transporting POC to deep ocean layers due to their large size and high sedimentation rates [28, 29]. However, the detailed structures of sinking particles and their decomposition processes during sedimentation from the surface to deep oceans remain relatively unexplored.

## Materials and methods

### Cruises and sampling strategy

**Figure 1:**
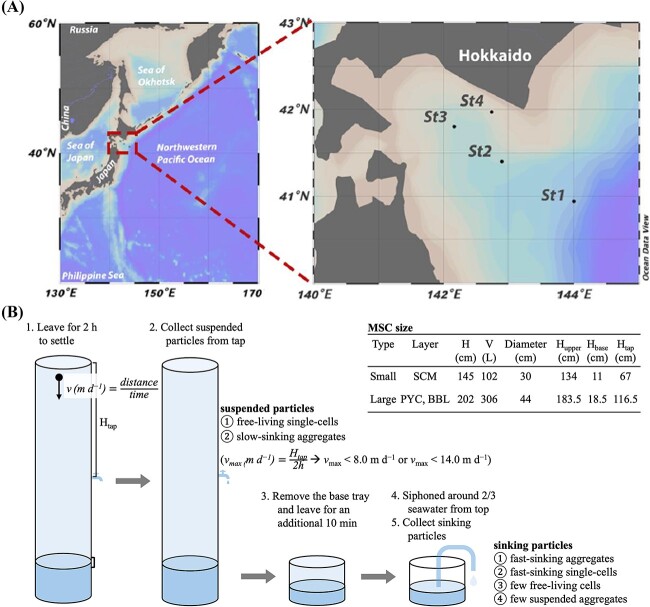
Illustration of sampling strategy. (A) the location of sampling sites visualized using ocean data view; (B) the schematic of sampling design with MSC. Two specifications of MSC were employed, and the detailed metrics were list in the table. The smaller MSC were deployed at SCM layer, while the larger one was used at both PYC and BBL.

Sampling was conducted at four stations (Fig. 1A) during the KS-21-4 (March 11–21, 2021) and KS-21-7 (May 3–11, 2021) cruises of the R/V *Shinsei-Maru* (JAMSTEC) in the Oyashio region off Hokkaido, Japan. Samples for DNA analysis were collected using MSCs at three depths: the subsurface chlorophyll maximum (hereafter SCM, 11–30 m depth), 10 m below the pycnocline (PYC, 65–250 m), and the bottom boundary layer (BBL, 289–1489 m; Table 1 and Table S1).

**Table 1:** Environmental variables of all samples in the Oyashio waters.

Sites	Cruise	Chl *a* (μg/L)	Layer^a^	Depth (m)	Temp (°C)	Sal (‰)	DO (l/L)	NO_3_^b^	NO_2_^b^	NH_4_^b^	PO_4_^b^	SiOH_4_^b^	POC flux^c^	PON flux^c^
St2	KS-21-4	1.17	SCM	17	1.37	32.93	7.62	17.94	0.24	< 0.1^d^	1.50	33.39	89.69	18.36
	(2021)		PYC	150	3.93	33.51	6.68	19.94	0.18	< 0.1	1.56	37.31	0	0
			BBL	1478	2.40	34.50	0.95	43.87	0.06	< 0.1	3.13	163.23	30.94	4.49
St3		5.4	SCM	30	1.63	32.89	7.93	14.63	0.32	< 0.1	1.34	29.34	153.40	30.84
			PYC	250	2.22	33.41	5.44	28.05	0.08	< 0.1	2.17	58.71	0	0
			BBL	1050	2.80	34.41	0.70	43.85	0.09	< 0.1	3.19	155.52	29.77	3.74
St4		11.93	SCM	11	0.47	32.57	8.28	9.60	0.18	< 0.1	1.08	23.39	228.71	49.71
			PYC	71	0.63	32.79	7.60	15.49	0.18	< 0.1	1.40	29.99	717.60	57.76
			BBL	289	1.76	33.47	6.12	19.32	0.23	< 0.1	1.62	37.47	86.96	12.74
St1	KS-21-7	1.51	SCM	30	2.19	33.02	7.80	9.30	0.16	1.05	1.03	2.95	385.34	64.79
	(2021)		PYC	65	4.54	33.09	7.20	23.73	0.40	1.47	2.03	41.12	274.79	53.54
St2		1.36	SCM	14	8.11	33.40	6.80	2.60	0.09	< 0.1	0.33	4.55	152.74	32.01
			PYC	65	5.52	33.49	6.31	12.03	0.19	0.56	1.04	17.95	103.29	22.10
St4		7.94	SCM	25	3.30	32.25	8.01	3.34	0.20	0.62	0.62	2.15	755.68	129.66
			PYC	90	1.93	33.18	6.69	20.48	0.27	0.63	1.77	36.57	208.48	39.90
			BBL	305	2.83	33.18	5.37	27.38	0.22	< 0.1	2.11	54.42	352.65	63.65

a SCM, Subsurface chlorophyll maximum; PYC, Pycnocline; BBL, Bottom boundary layer

b Macronutrient unit, μmol/L

c PON and POC flux, mg/m^2^/d

d lower than the detection limit (0.1 μmol/L)

Suspended particles were collected from tap at the center of the MSC, and sinking particles were collected from the base of the MSC. Since the base fraction contained some suspended particles, after detaching the upper part of the MSC, it was allowed to settle for an additional 10 min, and approximately two-thirds of the seawater was siphoned off to further reduce the suspended particles (Fig. 1B). The sample seawater was then filtered using a 0.8 μm pore-size cellulose acetate membrane (47 mm diameter, Millipore) under gentle vacuum (< 0.013 MPa). The filters were stored in 1.5-ml cryotubes pre-filled with 600 μl of buffer RLT Plus (Qiagen), 6 μl of 2-mercaptoethanol (Sigma-Aldrich), and 0.2 g of glass beads; the samples were flash-frozen in liquid N_2_ and stored at −80°C until analysis on land.

Note that the distinction of suspended and sinking particles is operational. Any particles (free-living single-cells and aggregates), remaining in the upper part of the MSC after 2 h settling, were regarded as suspended particles. Because we collected suspended particles from the tap located near the center of the MSC, suspended particles include not only non-sinking particles but also those sinking speed at up to 8.0 m d^−1^ (for small MSC) or up to 14.0 m d^−1^ (for large MSC, Fig. 1B). The sinking particles refer to aggregates, and fast-sinking single-cells that were recovered from the bottom tray of the MSC after 2 h settling.

### Measurements of environmental variables

Vertical profiles of temperature, dissolved oxygen (DO), salinity, chlorophyll *a* (Chl *a*) fluorescence, photosynthetically available radiation (PAR), and turbidity were measured at each site using an SBE 911-plus CTD system (Seabird Electronics, Bellevue, WA, USA; Fig. S1). Seawater samples for Chl *a* and macronutrient analysis were collected using Niskin bottles attached to a CTD-Carousel multiple-sampler system. Detailed procedures for Chl *a* and macronutrient analyses have been described by Fukuda et al. (2016) [30]. Briefly, samples for Chl *a* concentration measurement were collected on GF/F filters and measured with a fluorometer (10-AU, Turner Designs) using the acidification method [31]. The concentrations of macronutrients (Si(OH)_4_, NO_2_, NO_3_, NH_4_, and PO_4_) were determined using a flow injection analyzer (AACSIII, Bran + Luebbe). The detection limits of the macronutrient measurements were defined as three times the deviation of the repeated measurements from the blank. Samples for POC and PON concentrations were collected from the sinking particles of MSC and analyzed using an elemental analyzer (Elemental Analyzer-IRMS; FLASH 2000, Thermo Fisher Scientific). The POC and PON sinking fluxes were calculated using the methods described by Giering et al. (2016) [32] and Yamada et al. (2024) [33].

### DNA extraction and 18S rRNA gene amplification and sequencing

AllPrep DNA/RNA Mini Kits (Qiagen) were used for DNA extraction. Sample vials were agitated thrice at 2500 rpm for 50 s using a homogenizer (μT-12, TAITEC) before proceeding with the manufacturer’s protocol (BBL samples at St1 and St2 in May failed to yield sufficient DNA for downstream analysis). For each sample, three PCR amplifications targeting the V4 region of the 18S rRNA gene were performed using the following primer pairs: forward E572F (5’-CYGCGGTAATTCCAGCTC-3′) and reverse E1009R (5’-AYGGTATCTRATCRTCTTYG-3′; 436 bp), as designed previously [34]. The PCR mixtures were prepared in a 25-μl reaction volume containing 1 × KAPA HiFi HotStart ReadyMix, 1 μM each of forward and reverse primers, and 2.5 ng of template DNA. The PCR cycle consisted of a pre-denaturation step at 98°C for 30 s, followed by 30 cycles of denaturation at 98°C for 10 s, annealing at 61°C for 30 s, and extension at 72°C for 30 s, and a final extension at 72°C for 5 min. The triplicate PCR products were mixed after purification using Agencourt AMPure XP beads (Beckman Coulter). Libraries were prepared using the Nextera XT index kit V2 and sequenced on an Illumina MiSeq platform using a 300 bp paired-end sequencing.

### Analysis of sequencing data

Raw sequencing data were preprocessed using QIIME2 (version 2021.11) [35]. Trimming of low-quality reads and primer sequences, merging of paired-end reads, dereplication, chimera removal, and amplicon sequence variant (ASV) inference were performed using denoising algorithms in DADA2 with default parameters [36]. A naïve Bayes classifier [37] trained on the PR2 database [38] (v 5.0.1, comprising nine ranks: domain, supergroup, division, subdivision, class, order, family, genus, species) with the E572F/E1009R primer set was used to assign taxonomy to the ASV sequences. The feature table of read counts and taxonomy assigned to ASVs was exported; singletons and those unannotated to the target supergroup ASVs were eliminated.

Next, we classified the ASVs into unicellular (protist) and other eukaryotes (Embryophyceae, Fungi, Metazoa, and Rhodophyta); the metazoan ASVs were recovered and independently analyzed. The trophic mode of protist ASVs was determined through the following steps (Fig. S2): (i) Non-Dinophyceae ASVs were first classified as mixotrophs and non-mixotrophs using the Mixoplankton Database [39] and data from Schneider et al. (2020) [40] (Table S2). (ii) The “non-mixotrophic” ASVs were then categorized into phototroph and heterotroph, with heterotroph further classified into phagotroph and parasite, based on the dominant ecological function of taxa as outlined in Sommeria-Klein et al. (2021) [41] and potential photosynthetic taxa listed in the PR2 database (Table S3). (iii) Trophic mode for Dinophyceae ASVs was assigned based on data from Schneider et al. (2020) [40], Mixoplankton Databases [39], and existing literature (Table S4); (iv) all protists ASVs that could not be categorized based on prior steps were labeled as “unknown”. Considering that taxonomy-based trophic modes classification can be challenging due to the wide variation among protists and the limitations of available databases, we combined multiple reliable sources to ensure the most accurate classification. We acknowledge that future research may further refine these categories.

### ASV-based diversity analyses

Statistical analyses were performed using R v.4.4.1 and Python v.3.9.13. Samples were rarefied to account for differences in library size, and rarefaction curves were used to investigate the degree of sample saturation by calling the “rrarefy” function in the vegan v.2.6.4 package [42]. Non-metric multidimensional scaling (NMDS) analysis of the dissimilarity in the microeukaryotic communities and the relationship between the suspended microeukaryote composition and environmental variables (NH_4_ was omitted because 11 out of 16 samples had concentrations below the detection limit; Table 1) were performed based on the Bray–Curtis dissimilarities with the rarefied abundance table (with the “envfit” function in vegan). Permutational multivariate analysis of variance (PERMANOVA) was used to assess the statistically significant dissimilarities. Alpha diversity indices (*Pielou’*s evenness and richness) of phototrophs and heterotrophs in each sample were evaluated by rarefying read numbers. Differences in alpha diversity between these two particle fractions were assessed using the Wilcoxon signed-rank exact test to determine statistical significance.

We assumed that sinking protists in the deep layer may have originated from direct sinking events from the SCM layer and/or colonization by free-living protists during sedimentation. To estimate the relative contributions of these sources to the sinking protists in the deep sea, we used the SourceTracker (v 2.0.1, http://github.com/biota/sourcetracker2) algorithm. This algorithm employs the Bayesian classification model and Gibbs sampling to predict the proportion of microbial communities from a given set of “source” communities that contribute to “sink” communities [43]. Using the protist ASV abundance table, sinking protists at SCM and suspended protists at PYC and BBL were considered as potential “sources”, while sinking protists at BBL acted as “sinks”. To provide a robust starting point for each “source” and minimize the risk of overfitting, SourceTracker was executed with default settings; the configuration of the algorithm is detailed in Knights et al. (2011) [43].

### Taxon-based differential abundance analysis

Differential analysis of protist genera between sinking and suspended particles was performed using DEseq2 v.1.38.3 [44]. Protist genera with an average relative abundance of less than 0.01% and those belonging to ambiguous classes (unassigned, Gyrista_X, Ciliophora_X, Cercozoa_X) and classes with low relative abundance (less than 0.2% on average) were excluded from further analysis. Protist genera with *log_2_* fold change >1.5 or < −1.5 and an adjusted *p*-value <0.01 determined using the Benjamini–Hochberg (BH) method was regarded as “significant”.

## Results

### Sequencing statistics and environmental conditions

A total of 3 913 663 18S rRNA gene (V4 region) paired-end reads were obtained from 32 suspended and sinking particles. After quality trimming, merging, and the removal of chimera and singleton, 2416 ASVs were generated. Among these, 171 ASVs (an average of 14.3% of total reads) were taxonomically assigned to metazoan, 22 ASVs (an average of 0.2%) to Fungi. Independent analysis of metazoan ASVs showed that most metazoan reads were annotated to copepods (79.0% ± 26.0%), including the genera *Oithona*, *Metridia*, and *Eucalanus* (Fig. S3). Metazoan and other non-protists ASVs were excluded. The remaining 2204 protist ASVs were subjected to downstream analyses, and their relative frequencies were normalized by rarefying the smallest number of sequences per sample (33 794 paired-end reads; Fig. S4).

The protist ASVs were systematically classified into four functional categories: phototrophs (412 ASVs), phagotrophs (812 ASVs), parasites (517 ASVs), and mixotrophs (54 ASVs). The remaining 406 ASVs that could not be assigned to any functional category were labeled as “unknown” (Fig. S5a–c, Tables S1 and S5), and 341 of these were affiliated with Dinophyceae (Fig. S5c). On average, phototrophs, phagotrophs, and “unknown” comprised most reads (33.5%, 33.1%, and 26.5% of relative frequencies, respectively), while parasites and mixotrophs represented smaller proportions (4.7% and 2.1%, respectively; Fig. S5b).

High Chl *a* concentrations were detected at St3 (5.4 μg/L) and St4 (11.9 μg/L) in March and at St4 (7.9 μg/L) in May. Relatively higher POC fluxes (153.4–755.7 mg C m^−2^ d^−1^) and PON fluxes (30.9–129.7 mg N m^−2^ d^−1^) were observed in these sites than in the other sites (89.7–385.3 mg C m^−2^ d^−1^ and 18.4–64.8 mg N m^−2^ d^−1^; Table 1).

### Microeukaryotic community composition

**Figure 2:**
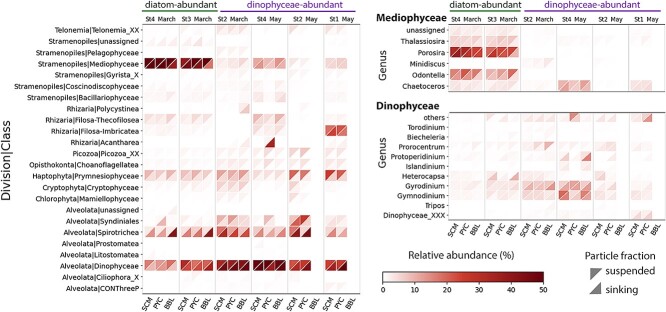
Overview of protistan class composition in Oyashio waters. Only classes with relative abundance greater than 1% in a single sample were retained. For the main classes (Mediophyceae and Dinophyceae), more detailed taxonomic composition is shown at the genus level; only the genera with relative abundance greater than 1% in a single sample are shown. The x-axis indicates different sampling depths. Here and throughout the paper, SCM stands for subsurface chlorophyll maximum layer, PYC for Pycnocline layer, and BBL for bottom boundary layer. Different triangle shapes represent different particle fractions.

Mediophyceae (a class of diatoms) dominated the microeukaryote community at St3 and St4 in March, representing over half of the protist 18S reads (52.0% ± 11.6%) in both sinking and suspended particles at SCM (Fig. 2). Due to the high Chl *a* concentration (> 5 μg/L) and the prevalence of diatoms, these samples were classified as “diatom-abundant”. Notably, the chain-forming diatoms *Porosira* and *Odontella* were the most prominent genera, even at depths exceeding 1000 m (Fig. 2). Dinophyceae were more abundant in other samples (37.2% ± 11.8%) compared to diatom-abundant samples (20.1% ± 9.6%); these samples are hereafter referred to as “Dinophyceae-abundant” samples. NMDS analysis revealed a significant difference in community composition between Dinophyceae-abundant and diatom-abundant samples (PERMANOVA, *P* < 0.001; Fig. S6a and Table S6). By May, Dinophyceae had become the dominant group in the sinking microeukaryotic communities, coinciding with peaks in POC/PON fluxes (Fig. 2, Fig. S6b–c, and Table 1). Additionally, prymnesiophytes, particularly *Phaeocystis*, were more abundant in suspended particles at SCM compared to their presence in sinking particles, where their abundance decreased with depth (Fig. 2).

### Microeukaryotic communities differ between suspended and sinking fractions

**Figure 3:**
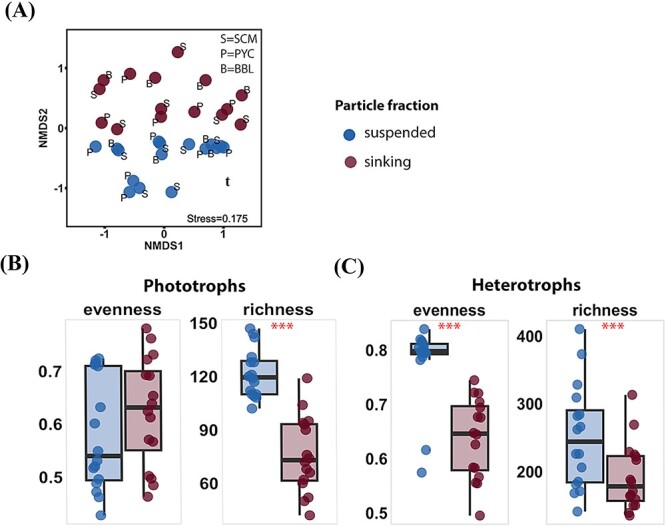
Comparison of microeukaryotic communities detected in different particle fractions. (A) NMDS based on bray–Curtis dissimilarities. Letters indicate depth: S=SCM, P=PYC, B=BBL. (B-C) Evenness and richness indices of phototrophs (B) and heterotrophs (C) groups pooled by particle fractions. Stars indicate significant differences in indices between these two particle fractions, with ^*^p < 0.05, ^*^^*^p < 0.01, ^*^^*^^*^p < 0.001 (Wilcoxon test).

The NMDS showed a significant separation in the ASV composition of the microeukaryotic community between sinking and suspended particles (PERMANOVA, *P* < 0.05; Fig. 3A and Table S6). The microeukaryotic community had a significantly lower richness (Wilcoxon test, *P* < 0.001) in sinking particles (Fig. S6d). Specifically, the richness of phototrophs was lower in sinking particles than that in suspended particles (Wilcoxon test, *P* < 0.001; Fig. 3B, and Fig. S6e). Heterotrophs exhibited significantly lower *Pielou*’s evenness (Wilcoxon test, *P* < 0.001) and richness (Wilcoxon test, *P* < 0.001) in sinking particles than that in suspended particles (Fig. 3C and Fig. S6f). The proportions of ASVs unique to the sinking particles (7.3% ± 3.1% for phototrophs and 20.3% ± 8.0% for heterotrophs) were lower than those unique to the suspended particles and shared in both particles (Fig. S7).

### Distribution pattern and vertical variation of phototrophs in sinking and suspended particle

**Figure 4:**
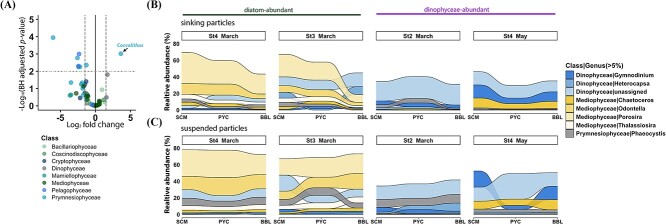
The distribution and variation of phototrophic genera in sinking and suspended particles. (A) Volcano plot showing different abundance of phototrophs between these two particles. The *log*2 fold change is plotted against -*log* (BH adjusted *p*-value), mainly at the genus level. Each dot represents a genus, color-coded based on their class information. Significantly distinct genera between these two particles were defined based on the | *log*2 fold change | > 1.5 and BH adjusted *p*-value <0.01. The *log*2 fold change >0 represents genera enriched in sinking particles, while *log*2 fold change <0 indicates genera enriched in suspended particles. (B, C) the vertical succession of sinking particle-associated (B) and suspended particle-associated (C) phototrophic genera. Only genera with relative abundance larger than 5% in a single sample are shown. The Dinophyceae genera assigned to “unknown” trophic mode were included, and stations without a BBL sample (St1 and St2 in may) were excluded from the analysis.

Differential abundance analyses revealed varying patterns in the relative abundance of phototrophic genera between sinking and suspended particles (Fig. 4A, Fig. S8a, and Table S7). Only *Coccolithus* (a genus of haptophyte) was significantly enriched in sinking particles (BH adjusted *p*-value <0.01; Fig. 4A). Most genera belonging to Prymnesiophyceae (9 out of 10 genera), Pelagophyceae (three out of three genera), and Mamiellophyceae (four out of four genera) were enriched in suspended particles, with seven genera showing statistically significant differences (BH adjusted *p*-value <0.01; Fig. 4A). No significant difference was observed in the phototrophic Dinophyceae genera between the two particle types, while some mixotrophic genera and genera with “unknown” trophic modes were more prevalent in sinking particles, with two genera showing statistical significances (BH adjusted *p*-value <0.01; Fig. S8b–c).

The composition of key phototrophic classes changed with depth in sinking particles, with the relative abundance of total phototrophs decreasing, especially in diatom-abundant samples (from 61.6% ± 11.2% at SCM to 31.2% ± 15.1% at BBL; Fig. S5d). The relative abundance of the dominant genera *Porosira* and *Odontella* displayed remarkable decreases in sinking particles while remaining consistently high in suspended particles along with increasing depth (Fig. 4B–C).

### Sinking pattern of heterotrophs

**Figure 5:**
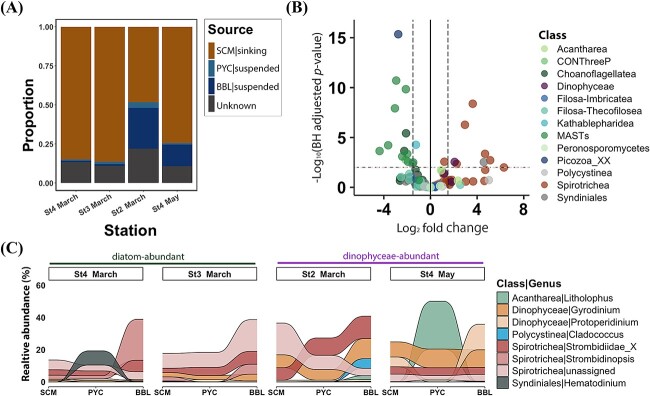
Sinking dynamics of heterotrophs. (A) Proportion estimates of the source for sinking heterotrophs at BBL using the Bayesian source tracking algorithm. Four sources include: Sinking heterotrophs at SCM, suspended heterotrophs at PYC, suspended heterotrophs at BBL, unknown. (B) Volcano plot depicting the different abundance of heterotrophs between sinking and suspended particles. The *lo*g2 fold change is plotted against -*log* (BH adjusted *p*-value), mostly at the genus level. Each dot represents a genus, color-coded based on their class information. Significantly distinct genera between these two particles were defined based on | *log*2 fold change | > 1.5 and BH adjusted *p*-value <0.01. The *log*2 fold change >0 indicates genera enriched in sinking particles, while *log*2 fold change <0 indicates genera enriched in suspended particles. (C) Vertical succession of sinking particle-associated heterotrophic genera. Only genera with relative abundance larger than 5% in a single sample are shown. Stations without a BBL sample (St1 and St2 in may) were excluded from the analysis.

The SourceTracker analysis indicated that most sinking heterotrophs at BBL likely originated from sinking particles at SCM (74.0% ± 22.2%; Fig. 5A). This value is comparable to that observed for phototrophs (75.0% ± 12.0%, Fig. S9). In contrast, relatively low proportions of heterotrophs were traced to suspended heterotrophs at PYC and BBL (3.5% ± 3.3% and 12.0% ± 17.0%, respectively). In addition, potential sources of small fractions of sinking heterotrophs were not identified by the algorithm (12.3% ± 4.0%). Consistently, large proportions of heterotroph ASVs were shared between SCM and PYC (49.0%) PYC and BBL (50.1%) (Fig. S10).

Heterotrophic genera showed distinct distribution patterns between the two particle types (Fig. 5B and Table S7). Notably, most Spirotrichea (a class of ciliates) genera were predominant in sinking particles (20 out of 24 genera), with seven of these showing statistically significant enrichment (BH adjusted *p*-value <0.01; Fig. 5B). Most heterotrophic Dinophyceae genera were relatively enriched in sinking particles (four out of five genera), with one genus being significantly enriched. In contrast, MAST lineages (MAST-1, MAST-2, MAST-7, and MAST-8), which encompass heterotrophic nanoflagellates, were primarily detected in suspended particles.

As the relative abundance of sinking phototrophs decreased, the relative abundance of heterotrophs increased with depth (Fig. S5d). The results showed a distinct vertical succession of sinking heterotrophs during sedimentation (Fig. 5C). Notably, the Spirotrichea genera, such as *Strombidinopsis*, were more prevalent in the deep ocean, as did heterotrophic Dinophyceae genera such as *Gyrodinium*. However, the genera *Litholophus* and *Hematodinium* were primarily observed at PYC and were rarely detected at BBL.

## Discussion

The combined use of MSC and DNA metabarcoding enabled us to characterize the microeukaryotic communities that contribute to the biological carbon sequestration processes during spring in the Oyashio waters. However, we acknowledge that metabarcoding results do not necessarily reflect the abundance of plankton cells, as variance in 18S gene copy number between species might have obscured the relationship between amplicon sequence counts and cell abundance. Nevertheless, previous studies have shown a strong correlation between the number of 18S gene copies and cellular biomass or biovolume in certain protists [45, 46]. Therefore, amplicon sequencing is considered to provide extensive and comprehensive taxonomic information about a microbial community as well as ecologically relevant indices of community composition. Additionally, the statistical results of differential abundance analysis should be carefully interpreted since the observed counts of ASVs are necessarily represented as compositional data but not absolute abundances.

We observed a clear separation of eukaryotic community composition between suspended and sinking particles (Fig. 3A). The lower community richness in sinking particles (Fig. S6d) was attributable to selective aggregation and sinking processes. Namely, a subset of the microeukaryotic taxa in suspended particles was incorporated into the aggregates, contributing to the sinking processes. Additionally, the selectivity for aggregation and sinking could be influenced by the physical and physiological properties of the phototrophs (size, shape, stickiness, and secretion of exopolymers), which can affect aggregate formation and sedimentation [47].

Prymnesiophytes, mamiellophytes, and pelagophytes, mostly belonging to pico- and nanoplankton (0.2–20 μm), were enriched in suspended particles (Fig. 2 and 4). Their small cell size likely led to a lower sinking velocity of individual cells [48, 49], making them more prone to being collected as suspended particles. Although these picophytoplankton groups were involved in sinking particles in the surface layer, they were rarely detected in the sinking particles in the deep layer, implying a higher tendency toward disaggregation or decomposition during the sinking process. This finding aligns with previous observations showing that prymnesiophyte-enriched particles exported from the euphotic zone are likely to disintegrate into suspended particles in the upper mesopelagic layer [14].

Diatoms are suggested to play a pivotal role in POC sedimentation in Oyashio waters [28, 29]. In this study, centric diatom genera such as *Porosira* and *Odontella*, belonging to microphytoplankton (20–200 μm) [50], were most prevalent in the diatom-blooming sites near coastal areas (Fig. 1 and 2). Diatoms are typically considered important contributors to BCP in various ecosystems, as they have relatively large cells, and their heavy silica shells serve as ballast for marine snow and fecal pellets [51, 52]. Previous observations of Oyashio water during spring showed that *Porosira* was the most dominant genus during the bloom period, contributing to 25% of the diatom carbon biomass [53]. In our study, *Porosira* was enriched in sinking particles at BBL (16.8 ± 10.5%; Fig. 2), indicating that the dominant diatom would be the significant contributor to vertical carbon export. However, the relative abundance of *Porosira* in sinking particles decreased sharply with depth, especially from PYC (250 m, 21.7%) to BBL (1050 m, 9.4%) at St3 in March (Fig. 2 and 4B). In contrast, the relative abundance of *Porosira* in suspended particles remained high at both PYC and BBL (Fig. 4C). This indicates that while dominant diatoms sink to deeper layers, they experience high attenuation within the sinking communities. These findings are in agreement with the results of previous field studies, which reported lower transfer efficiencies in the diatom-blooming sites than in the non-blooming sites [54–56]. Sinking aggregates composed of diatoms in blooming sites typically exhibit lower sinking velocity than that in non-blooming sites due to their lower density associated with higher porosity. Of note, the lower sinking speed increases their susceptibility to grazing by copepods and heterotrophic protists [5, 54]. Furthermore, the transparent exopolymer particles (TEPs) produced by diatoms [57], which facilitate aggregation, can reduce the sinking velocities of aggregates owing to their low density [58]. However, at St4 in May, the decline in *Porosira* from PYC (71 m, 28.3%) to BBL (289 m, 23.7%) was less pronounced, potentially attributable to the shallower depth of the BBL at this station. This shallower depth may have constrained the duration available for substantial compositional alterations to occur within the sinking particle communities during settling.

Dinophyceae were more abundant in sinking particles and showed stable relative abundance with depth (Fig. 4B and 5C). Dinophyceae are generally more carbon-dense than diatoms, as evidenced by their carbon-to-cell volume ratio [59]; compared to diatoms, their sinking can sequester more carbon per volume of particles. Dinophyceae can sank rapidly even as individual cells (~27.5 m d^−1^) [60], and they can form the massive carbon-rich and thick-walled resting cysts during Dinophyceae blooms [61]. These characteristics renders them more resistant to degradation, contributing to the intense sedimentation of organic matter. Moreover, among plankton, dinoflagellates are adapted to various environments, including pelagic and benthic habitats, with a wide range of trophic strategies and prevalence of mixotrophy [62, 63]. However, due to the limitations of the current mixotrophic database, many dinoflagellates in our study were assigned an “unknown” trophic mode. Cohen et al. (2021) [64] demonstrated that dinoflagellates have the capacity to alter metabolic functionality, which is characterized by nutrient recycling and phagotrophy, in the mesopelagic zone with no associated change in community assemblage. The high trophic diversity and adaptability of the dinoflagellates indicate that Dinophyceae genera with “unknown” trophic mode identified in this study are more likely to adapt to the deep-sea habitat by changing their trophic strategies to heterotrophic, reducing the susceptibility to degradation. Thus, Dinophyceae can act as important contributors to BCP in Oyashio waters, as indicated by previous studies across various regions [17, 65, 66]. This is also supported by the result of vector fitting analysis, which showed a positive correlation between Dinophyceae abundance and POC/PON fluxes (Fig. S6b–c).

In contrast to phototrophs, heterotrophs in sinking particles in the deep ocean can come from multiple sources, including origin from the surface layers along with the formation of sinking aggregates or continuous colonization from free-living organisms during sinking. Therefore, identifying the potential origins of sinking heterotrophs in the deep ocean may be of primary importance for understanding their function in the carbon export process. The source tracking analysis results showed that the heterotrophs associated with sinking particles in the deep layers are mainly derived from the surface with sinking aggregates (Fig. 5A). Additionally, the source tracking analysis is unable to differentiate between *in situ* colonization and the repackaging of disaggregated sinking particles in the deep layer. This constraint may explain the prediction that ~21% of phototrophs in the BBL originate from deep-layer colonization (Fig. S9), which may result from the repackaging or disaggregation of surface-sourced sinking phototrophs. Thus, the contribution of the SCM source for heterotrophs may be underestimated. In short, these results collectively suggest that sinking particles play a role in the transfer of heterotrophic microeukaryotes from the surface to the deep ocean layers, as previously reported for heterotrophic bacteria [67, 68]. Notably, ~12% of sinking heterotrophs at the BBL were classified as “unknown” source, likely due to several factors. First, the BBL samples, were influenced by the benthic communities as these were collected at 10 m above the seafloor where we detected high turbidity (Fig. S1). Second, horizontal water movement may have introduced heterotrophs from other areas, considering the high heterogeneity of deep-sea eukaryotic communities [69]. Lastly, the disaggregation of sinking particles promoted vertical connectivity between different depths [67], complicating SourceTracker’s ability to identify specific sources, resulting in the “unknown” classification.

The composition of heterotrophs significantly differed between sinking and suspended particles, with a lower richness and evenness in sinking particles than in suspended particles (Fig. 3C, 5B, and Fig. S6d). These results suggest that specific protist groups were enriched in sinking particles. The most prominent heterotroph groups enriched in sinking particles included Spirotrichea and heterotrophic Dinophyceae (Fig. 5B–C). These protists are active consumers of phototrophs, including chain-forming diatoms [70, 71], suggesting that they contribute to the decomposition and transformation of diatom-enriched aggregates in Oyashio waters.

The MAST groups of heterotrophs were more abundant in the suspended than in the sinking particles. In addition, they were more abundant in the surface layers than in deeper layers, which is consistent with previous observations [72]. The MAST groups include consumers of heterotrophic bacteria and picoeukaryotes in the water column [73, 74] and parasites of diatoms in sediments (e.g. MAST-6) [75]. Considering their low abundance in sinking particles, it is less likely that the MAST groups in suspended particles collected from the deeper layers of the Oyashio region were transported from the upper layer. MAST groups prefer a free-living, suspended lifestyle and consume picoeukaryotes and bacteria. The distinct occurrences of heterotrophs in sinking and suspended particles reflect the differences in lifestyle and feeding habits [76].

Our study is the first to document the differences in microeukaryotic communities between suspended and sinking particles at a molecular taxonomic resolution in the Oyashio region. We highlighted an unexpectedly significant contribution of heterotrophic lineages such as Spirotrichea and heterotrophic Dinophyceae to the transformation processes of sinking particles, presumably by consuming phototrophic cells. The depth-dependent attenuation of the relative abundance of phototrophs was more prominent in diatom-abundant conditions than in Dinophyceae-abundant ones, implying that diatoms are more rapidly consumed by heterotrophs. This result indicates that the magnitude of sinking POC flux attenuation is influenced by dominant plankton (diatoms vs. Dinophyceae) and their consumers, which comprise the microbial consortia of sinking particles. This raises new questions about the impact of prey–predator interactions of microeukaryotes, which are potentially highly selective [77], on regulating POC flux attenuation with depth. In conclusion, our results contribute to understanding the relationship between microeukaryotic assemblages and their vertical export. However, as our dataset only includes 18S rRNA gene metabarcoding, other proxies of microeukaryotic abundance and activity such as microscopic cell counts, 18S rRNA metabarcoding, and metatranscriptomic data would be required to provide a more comprehensive understanding of biological processes of sinking aggregates.

## Supplementary Material

Supplementray_meterial_ycae136

tables_s1_to_s5_and_s7_ycae136

## Data Availability

All raw sequencing data for 18S rDNA amplicons are available at the DDBJ Sequence Read Archive under accession number DRA015710. All codes associated with this study are freely available at the GitHub repository: https://github.com/yosei-yung/oyashio-marinesnow-protists.
