# Supplementary material for: Taxon-specific contributions of microeukaryotes to biological carbon pump in the Oyashio region
Source: ISME Commun. 2024 Nov 4;4(1):ycae136. doi: 10.1093/ismeco/ycae136 (PMC11575449; doi:10.1093/ismeco/ycae136)
Supplement: Supplementray_meterial_ycae136 [file supplementray_meterial_ycae136.docx]

**Suppementray Material for**

**Taxon-specific contributions of microeukaryotes to biological carbon pump in the Oyashio region**

Qingwei Yang^1^, Yanhui Yang^2^, Jun Xia^1^, Hideki Fukuda^2^, Yusuke Okazaki^1^, Toshi Nagata^2^, Hiroyuki Ogata^1^, Hisashi Endo^1*^

^1^Institute for Chemical Research, Kyoto University, Uji, Kyoto, Japan

^2^Atmosphere and Ocean Research Institute, The University of Tokyo, Chiba, Japan

****Corresponding authors***:

H. Endo, E-mail: endo@scl.kyoto-u.ac.jp, Phone: +81-774-38-32

**This supplementary file includes:**

Supplementary Figs. S1-S10

Supplementary Table S6

Legends of Supplementary Tables S1-S5 and S7

Supplementary References

**
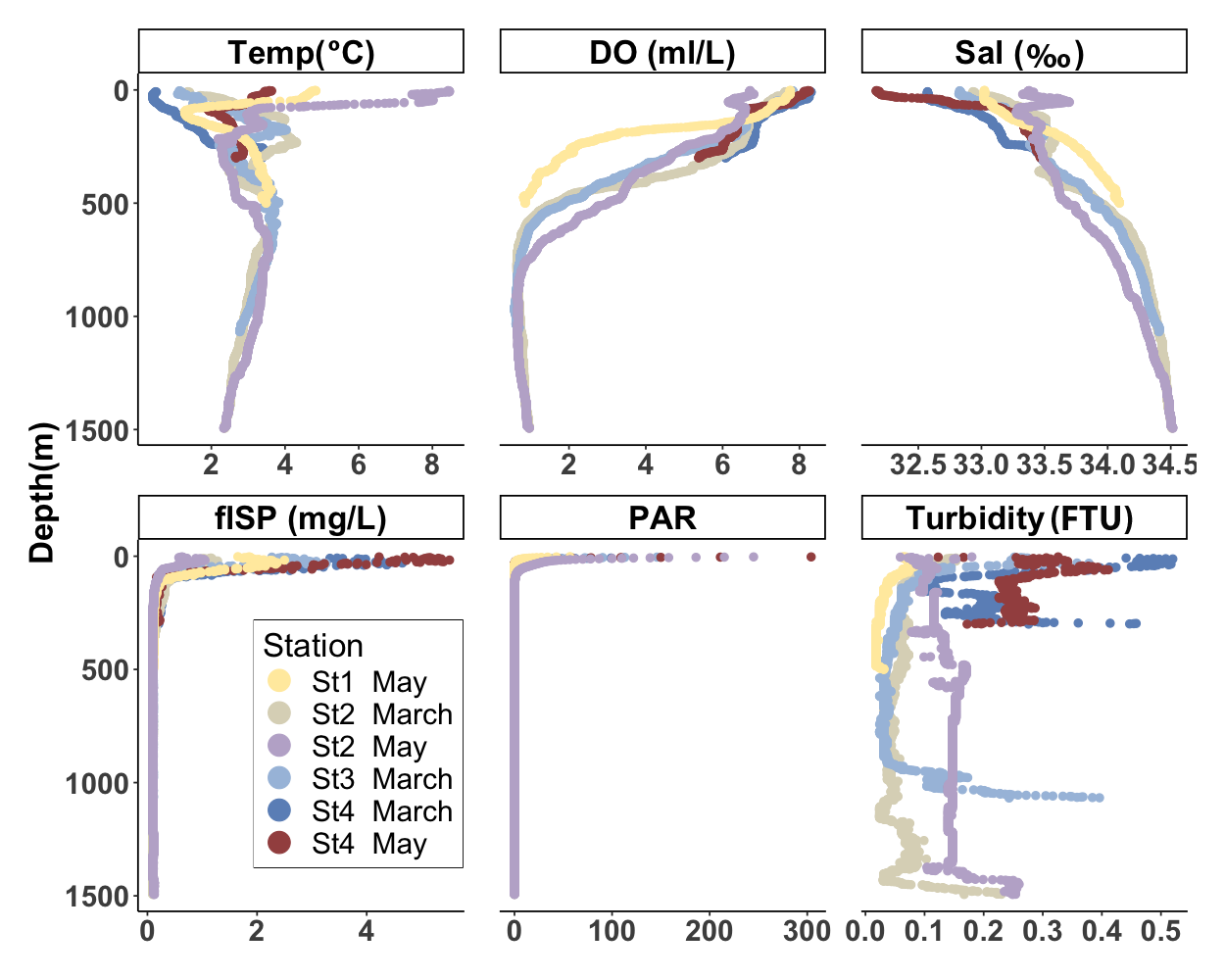
**

Figure S1 Profiles of water column properties as measured using a CTD profiler. Sensors measured Temp, DO, Sal, chlorophyll fluorescence (flSP, mg/L), photosynthetically active radiation (PAR), and turbidity.


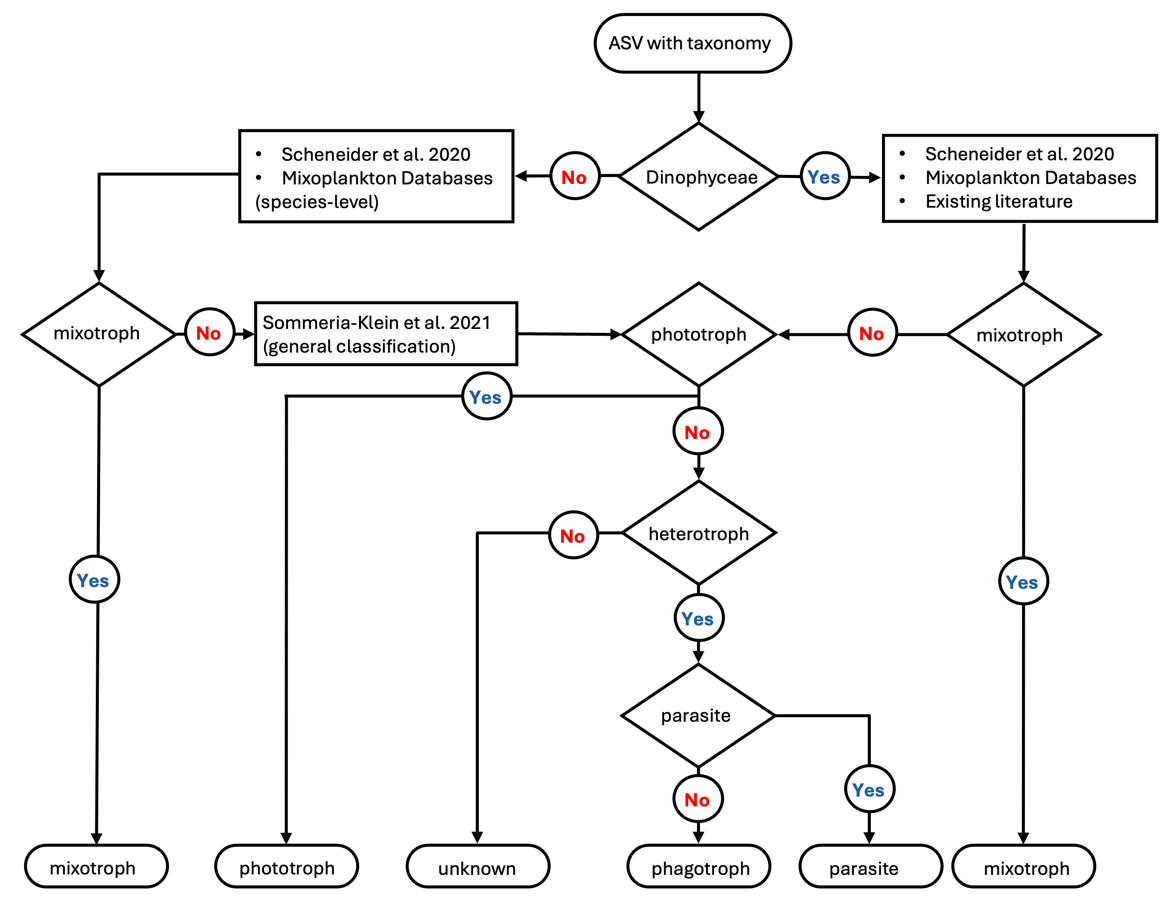


Figure S2 Schematic diagram of the classification of the trophic mode


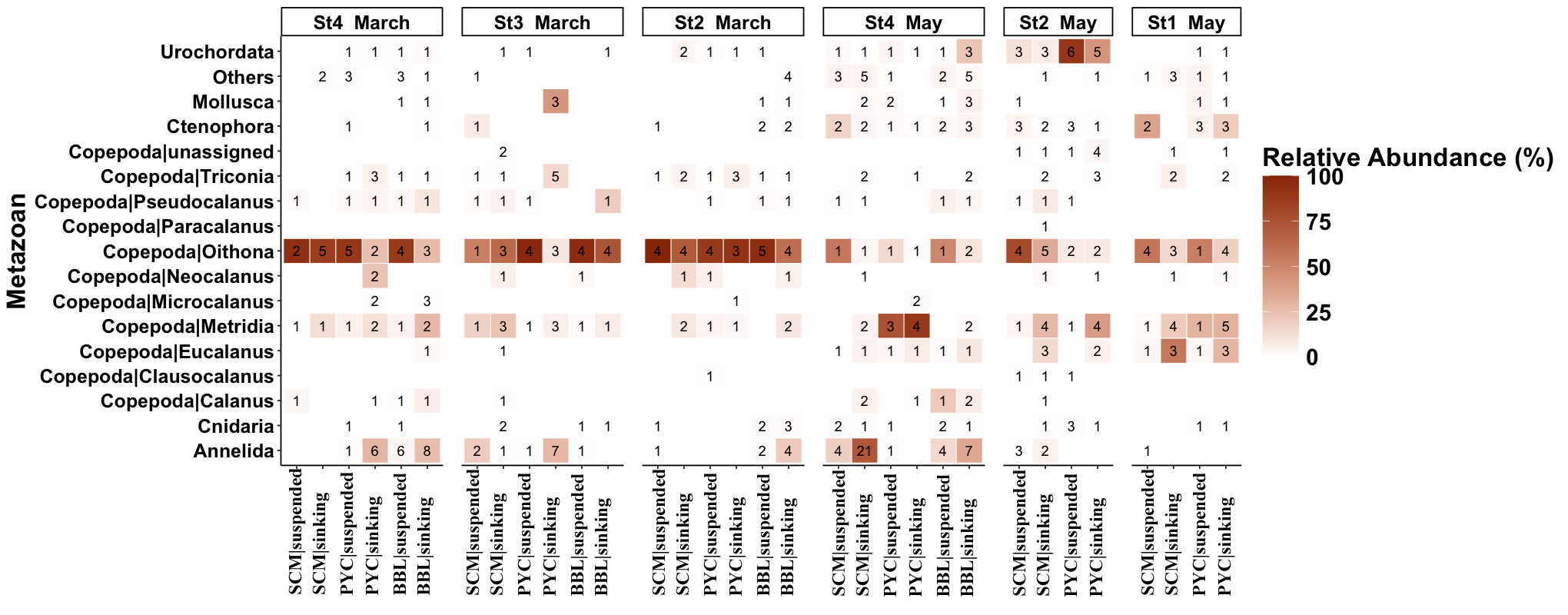


Figure S3 Metazoan composition in Oyashio waters, with colors indicating relative abundance and numbers indicating different ASVs.


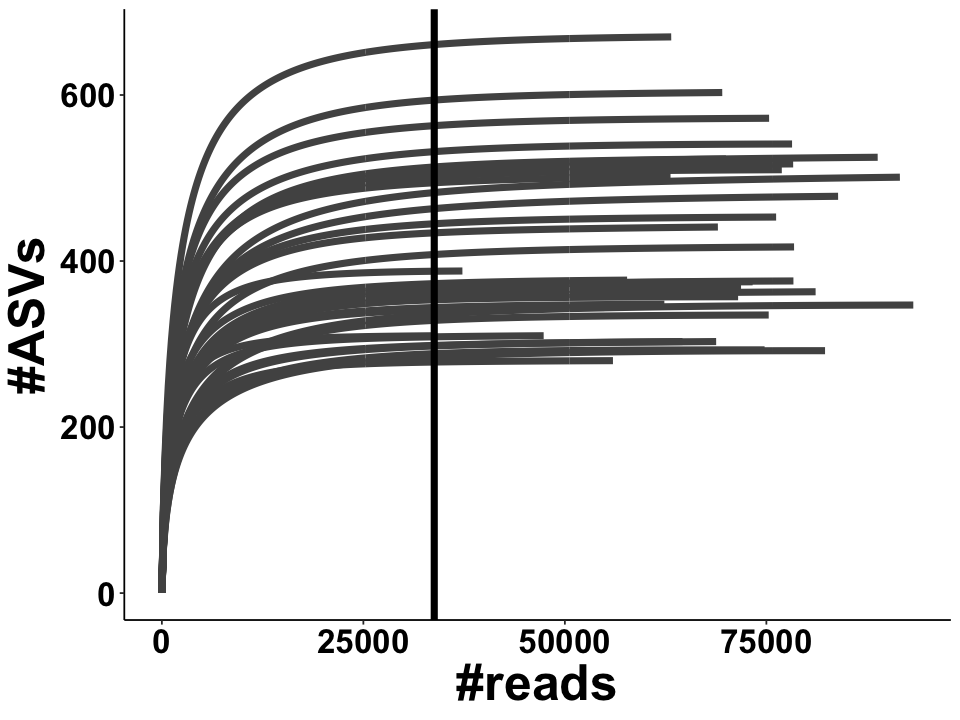


Figure S4 Rarefaction curves of the ASV numbers for each sample. The vertical black line represents the smallest number of sequences per sample (33,794 reads).


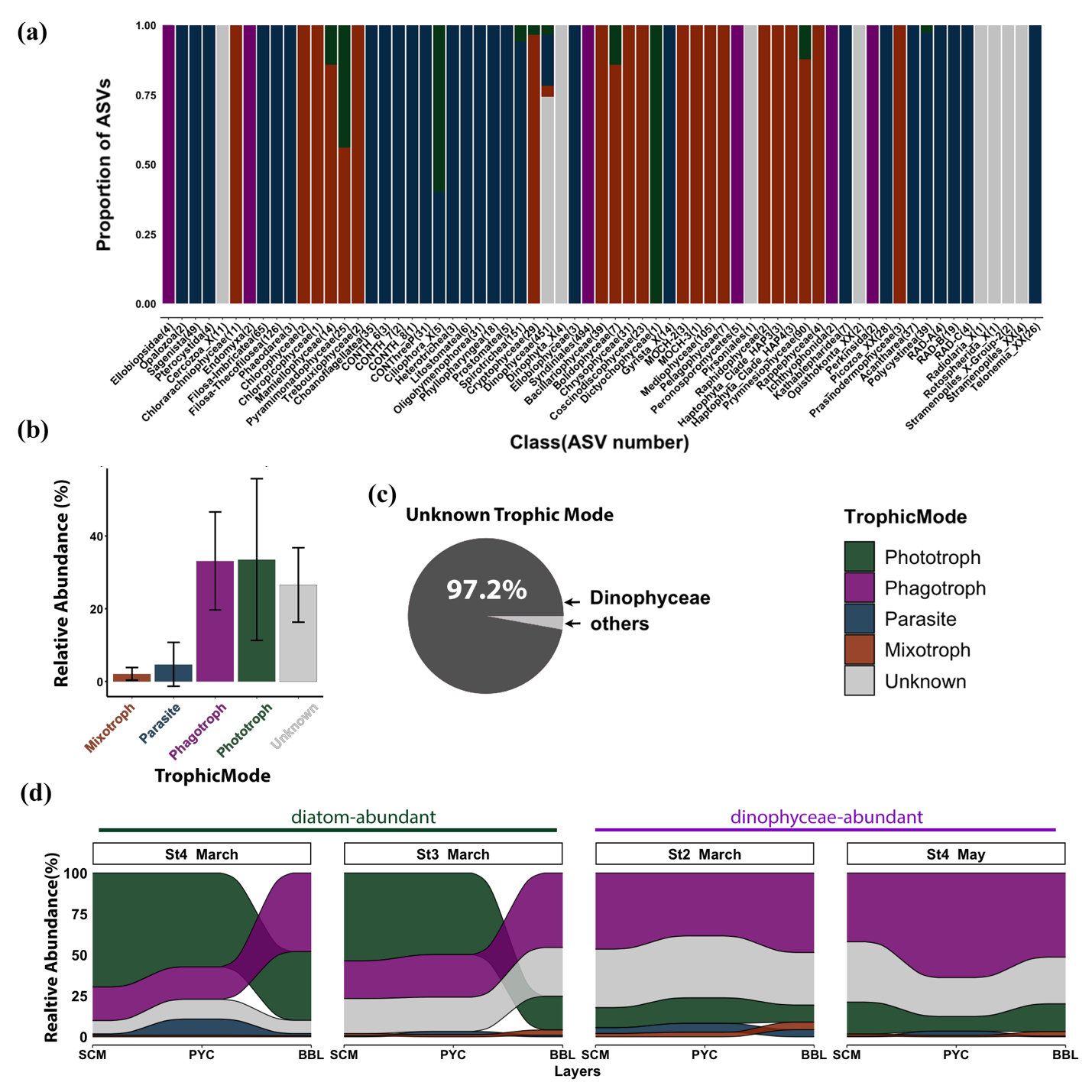


Figure S5 Distribution and variation of ASVs across trophic mode groups: (a) proportion of different trophic mode ASVs among classes. The x-axis represents the classes and the number in brackets indicates the number of ASVs in each class; (b) mean ± SD of relative frequencies of different trophic mode groups; (c) composition of the 'unknown' trophic group; (c) vertical variation of different trophic mode groups in sinking particles. Stations without a BBL sample (St1 and St2 in May) were excluded from the analysis.


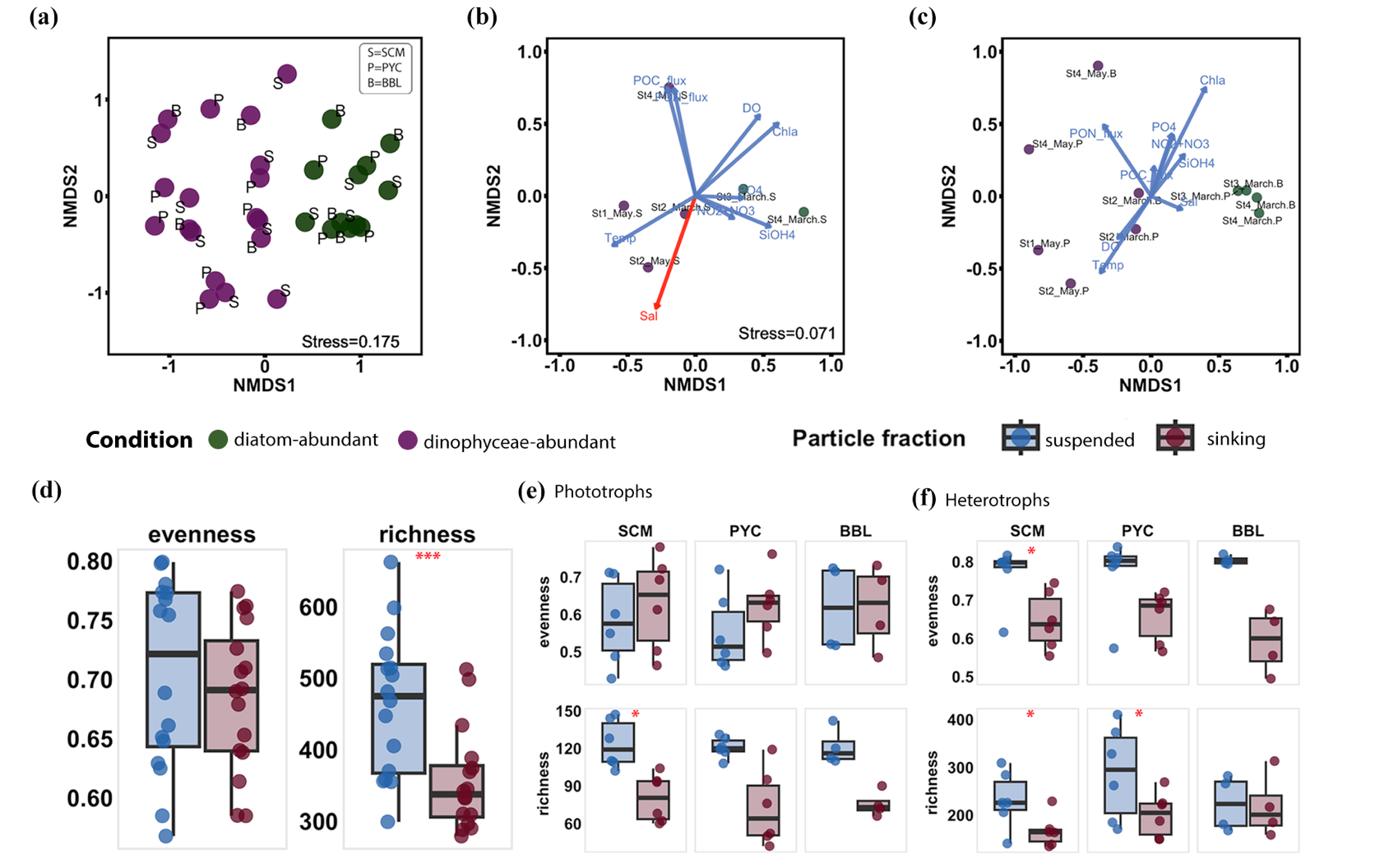


Figure S6 Comparison of microeukaryotic communities. (a) NMDS of Bray-Curtis dissimilarities. The samples were pooled by two conditions: diatom-abundant (red) and dinophyceae-abundant (purple) conditions. Letters indicate depth: S=SCM, P=PYC, B=BBL; (b-c) NMDS plot of microeukaryote protists in suspended particles for SCM (b) and PYC&BBL (c), including ten fitted environmental variables: total nitrogen (NO_2_ + NO_3_), phosphate (PO_4_), silicate (Si(OH)_4_), temperature (Temp), dissolved oxygen (DO), salinity (Sal), chlorophyll concentration (Chl *a*), and PON and POC fluxes. Variables with *p* < 0.05 are marked in red. Green points represent the diatom-abundant samples, and purple points represent the Dinophyceae-abundant samples; (d-f) Evenness and richness indices of microeukaryotic communities by particle fraction (d), and for phototrophs (e) and heterotrophs (f), categorized by both particle fraction and depth. Stars indicate significant differences in indices between these two particle fractions, with * *p* < 0.05, ** *p* < 0.01, *** *p* < 0.001 (Wilcoxon test).


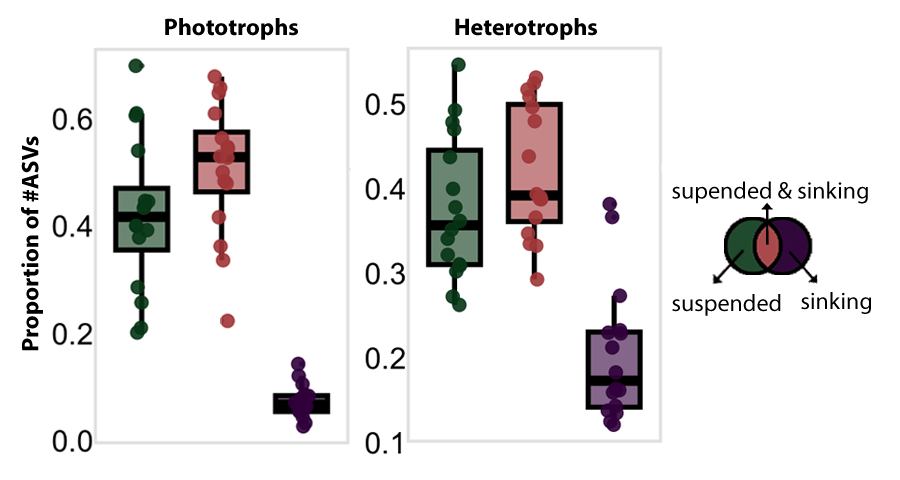


Figure S7 Box plots summarizing the proportions of ASVs that are unique to sinking particles (purple), unique to suspended particles (dark green), and shared in both particles (red).


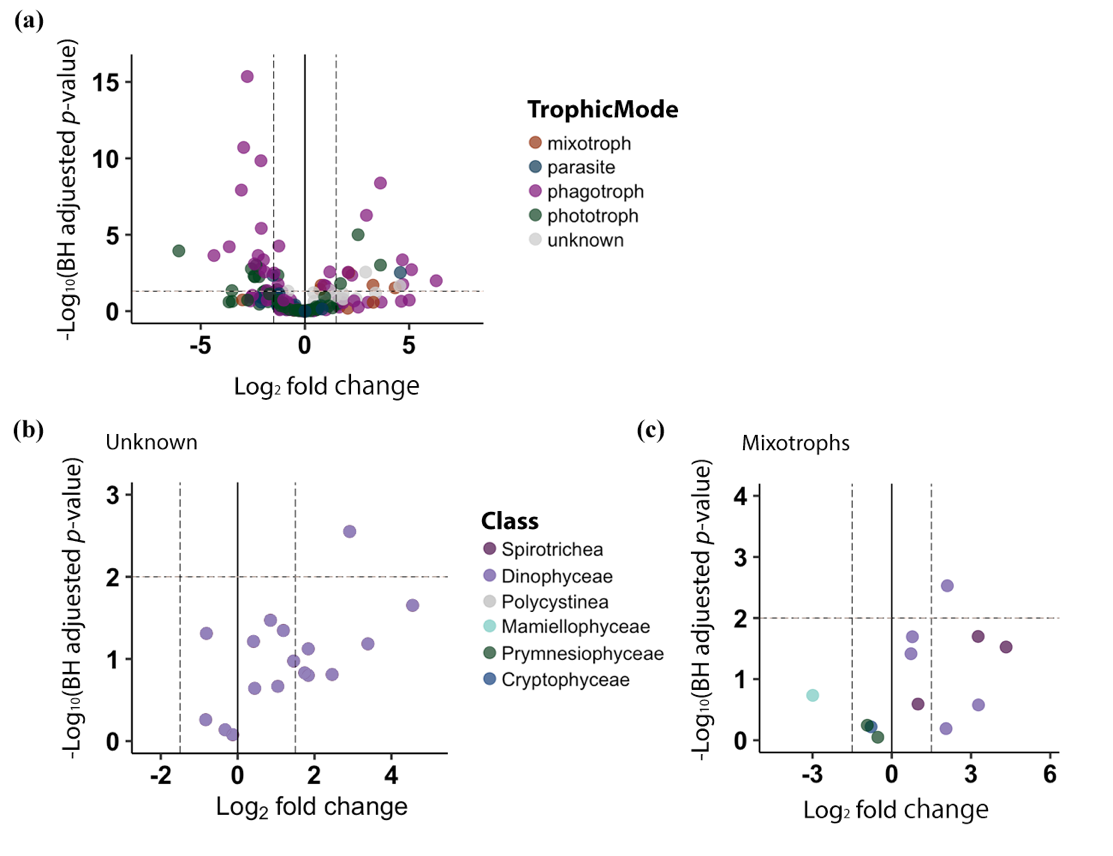


Figure S8 The volcano plot depicts the different abundance of different trophic mode (a), “unknown” (b) and mixotrophic(c) groups between sinking and suspended particles. *Log_2_* fold change is plotted against -*log* (BH adjusted *p*-value) mostly at the genus level. Each dot represents a genus, color-coded based on their class information. Significantly distinct genera between these two particles were defined based on the absolute of *log_2_* fold change >1.5 and BH adjusted *p*-value < 0.01. *Log_2_* fold change > 0 represent genus enriched in sinking particles, while *log_2_* fold change < 0 indicates genus enriched in suspended particles.


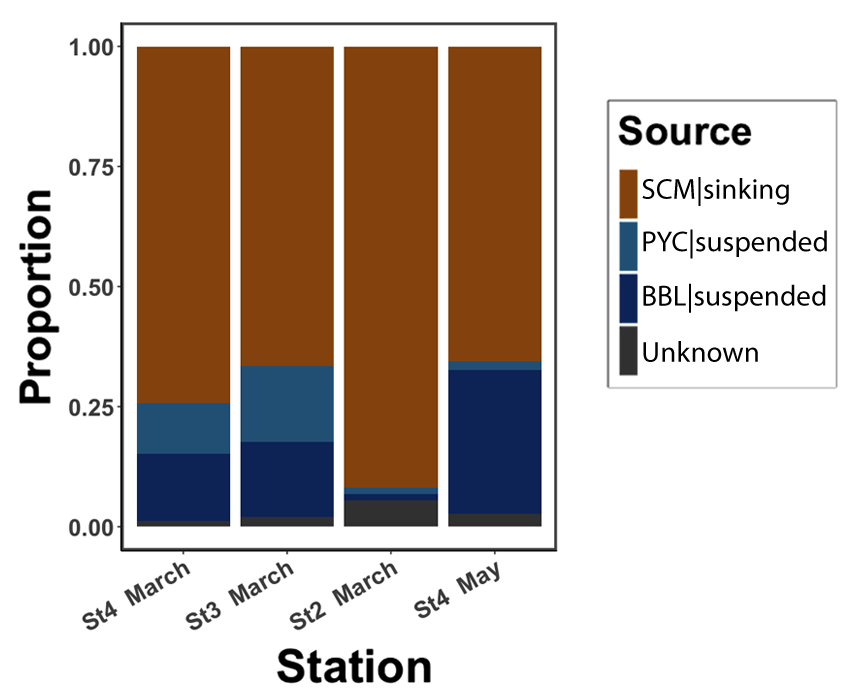


Figure S9 Proportion estimates of the source for sinking phototrophs at BBL using the Bayesian source tracking algorithm. Four sources include: sinking phototrophs at SCM, suspended phototrophs at PYC, suspended phototrophs at BBL,and Unknown.


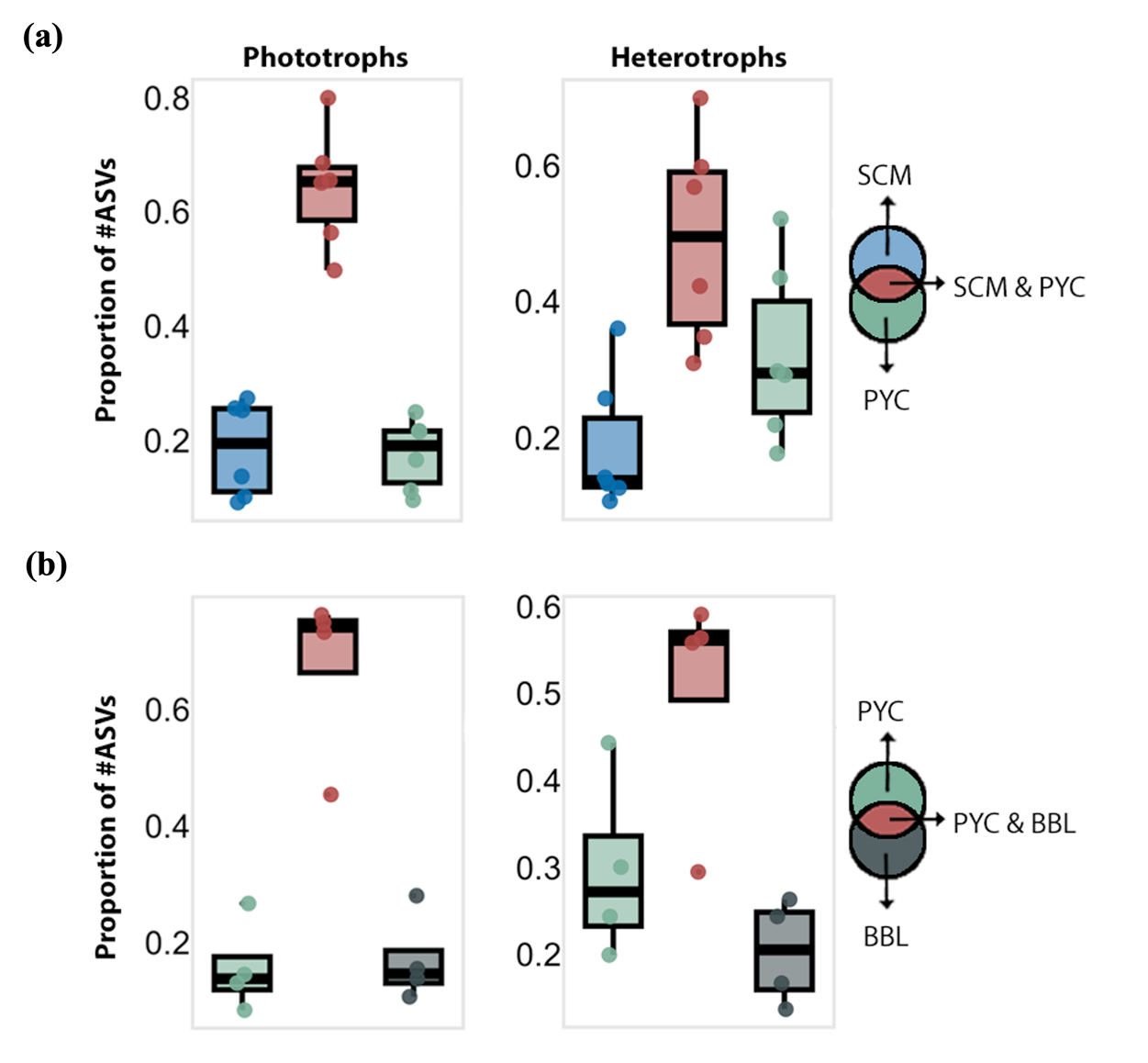


Figure S10 Box plots summarizing the proportions of unique and shared ASVs between different depths. The comparisons between SCM and PYC are shown in the upper panels, and those between PYC and BBL are shown in the lower panels. The results of phototrophs and heterotrophs are presented in the left and right panels, respectively.

Table S6 PERMANOVA results

| Source | Df^a^ | SumOfSqs^b^ | R2^c^ | F^d^ | Pr(>F)^e^ |
| --- | --- | --- | --- | --- | --- |
| ﻿﻿As.factor(particle types:suspended & sinking) | 1 | 0.5265432 | 0.09603109 | 3.186982 | 0.0032 |
| Residual | 30 | 4.9565055 | 0.90396891 | NA | NA |
| ﻿Total | 31 | 5.4830487 | 1.00000000 | NA | NA |
| ﻿As.factor(condition:diatom-dominant & dinophyceae-dominant) | 1 | 1.372062 | 0.2502371 | 10.01265 | 1e-04 |
| ﻿Residual | 30 | 4.110986 | 0.7497629 | NA | NA |
| ﻿Total | 31 | 5.483049 | 1.0000000 | NA | NA |

^a^ Df: stands for degrees of freedom

^b^ SumOfSqs: stands for sum of squares

^c^ R2: coefficient of determination

^d^ F: F-statistic

^e^ Pr(>F): *p*-value associated with the *F*-statistic

Table S1 Detail sampling information and summary statistics of the 18S sequencing data processing

Table S2 Identifies potential mixotrophs using the Mixoplankton Database and data from Schneider *et al.* (2020)

Table S3 The trophic mode assigned to the major protistan groups based on their known dominant ecological function with reference to the PR2 database, Sommeria-Klein *et al.* (2021) and other studies compiled in our study

Table S4 The trophic mode assigned to dinoflagellata (excluding Syndiniales class) with reference to Mixoplankton Database, Schneider *et al.* (2020) and other studies compiled in our study

Table S5 Complete metadata table for protists ASVs in this study

Table S6 The output of DEseq2 results and the ASVs number of each genus

**Supplementary reference**

Akselman, R., R. M. Negri, and E. Cozzolino. 2014. Azadinium (Amphidomataceae, Dinophyceae) en el Atlántico Sudoccidental: Observaciones in situ y satelitales. Rev. Biol. Mar. Oceanogr. 49: 511–526. doi:10.4067/S0718-19572014000300008

Bråte, J., R. Logares, C. Berney, D. K. Ree, D. Klaveness, K. S. Jakobsen, and K. Shalchian-Tabrizi. 2010. Freshwater Perkinsea and marine-freshwater colonizations revealed by pyrosequencing and phylogeny of environmental rDNA. ISME J. 4: 1144–1153. doi:10.1038/ismej.2010.39

Cavalier-Smith, T., and E. E.-Y. Chao. 2003. Phylogeny and Classification of Phylum Cercozoa (Protozoa). Protist 154: 341–358. doi:10.1078/143446103322454112

Díaz, P. A., C. Molinet, M. Seguel, M. Díaz, G. Labra, and R. I. Figueroa. 2018. Species diversity and abundance of dinoflagellate resting cysts seven months after a bloom of Alexandrium catenella in two contrasting coastal systems of the Chilean Inland Sea. Eur. J. Phycol. 53: 410–421. doi:10.1080/09670262.2018.1455111

Elshanawany, R., and K. A. F. Zonneveld. 2016. Dinoflagellate cyst distribution in the oligotrophic environments of the Gulf of Aqaba and northern Red Sea. Mar. Micropaleontol. 124: 29–44. doi:10.1016/j.marmicro.2016.01.003

Gavelis, G. S., S. Hayakawa, R. A. White III, T. Gojobori, C. A. Suttle, P. J. Keeling, and B. S. Leander. 2015. Eye-like ocelloids are built from different endosymbiotically acquired components. Nature 523: 204–207. doi:10.1038/nature14593

Gómez, F., P. López-García, A. Nowaczyk, and D. Moreira. 2009. The crustacean parasites Ellobiopsis Caullery, 1910 and Thalassomyces Niezabitowski, 1913 form a monophyletic divergent clade within the Alveolata. Syst. Parasitol. 74: 65–74. doi:10.1007/s11230-009-9199-1

Johnson, M. D., and D. J. Beaudoin. 2019. The genetic diversity of plastids associated with mixotrophic oligotrich ciliates. Limnol. Oceanogr. 64: 2187–2201. doi:10.1002/lno.11178

Ragan, M. A., C. L. Goggin, R. J. Cawthorn, and others. 1996. A novel clade of protistan parasites near the animal-fungal divergence. Proc. Natl. Acad. Sci. 93: 11907–11912. doi:10.1073/pnas.93.21.11907

Schneider, L. K., K. J. Flynn, P. M. J. Herman, T. A. Troost, and W. Stolte. 2020. Exploring the Trophic Spectrum: Placing Mixoplankton Into Marine Protist Communities of the Southern North Sea. Front. Mar. Sci. 7.

Sommeria-Klein, G., R. Watteaux, F. M. Ibarbalz, J. J. Pierella Karlusich, D. Iudicone, C. Bowler, and H. Morlon. 2021. Global drivers of eukaryotic plankton biogeography in the sunlit ocean. Science **374**: 594–599. doi:10.1126/science.abb3717

Yamaguchi, A., H. Kawamura, and T. Horiguchi. 2006. A further phylogenetic study of the heterotrophic dinoflagellate genus, Protoperidinium (Dinophyceae) based on small and large subunit ribosomal RNA gene sequences. Phycol. Res. **54**: 317–329. doi:10.1111/j.1440-1835.2006.00438.x

Yamaguchi, A., S. Yoshimatsu, M. Hoppenrath, K. C. Wakeman, and H. Kawai. 2016. Molecular Phylogeny of the Benthic Dinoflagellate Genus Amphidiniopsis and its Relationships with the Family Protoperidiniaceae. Protist **167**: 568–583. doi:10.1016/j.protis.2016.09.003
